# Supplementary material for: Urinary Neuropilin-1: A Predictive Biomarker for Renal Outcome in Lupus Nephritis
Source: Int J Mol Sci. 2019 Sep 17;20(18):4601. doi: 10.3390/ijms20184601 (PMC6769814; doi:10.3390/ijms20184601)
Supplement: Supplementary file 1 [file ijms-20-04601-s001.pdf]

International Journal of
Molecular Sciences

1. SI Materials and Methods

1.1. Patients

Renal involvement was documented by either a total urinary protein level ≥0.5 g/day, an increment of serum creatinine levels of more than 0.5 mg/dL, or the presence of active sediment by microscopic examination; more details in supporting information. Renal flares at inclusion were categorized as nephritic (≥30% increase in serum creatinine or decrease in estimated glomerular filtration rate (eGFR) by 10% with an active urine sediment, irrespective of changes in proteinuria) or proteinuric (reproducible doubling of the Upro-Ucre ratio >0.9 mg/mg after complete response or reproducible doubling of the Upro-Ucre ratio to >1.8 mg/mg after partial response) [37]. The renal samples were classified according to the histological types of LN using the ISN/RPS 2003 classification [38]. Renal biopsies were examined by light and immunofluorescence microscopy and categorised according to the International Society of Nephrology/Renal Pathology Society Classification [ISN/RPS] [37] and rated for activity (AI) and chronicity (CI) [36].

All patients were treated with IV methyl-prednisolone (500 mgx3), followed by a tapering dose of oral prednisone along with at least a 24-month course of oral mycophenolate mofetil [39]. In the prospective study, urine and blood samples were also obtained on a three-monthly basis for 1 year. All patients also received supportive treatment with angiotensin-converting enzyme inhibitor or angiotensin-receptor blocker and statin-lowering therapy. In the prospective study, patients were followed up every three months for one year. At each time point, urine and blood samples were obtained.

1.2. Study Design

First, urinary NRP-1 levels were measured in 45 patients with active LN prior to renal biopsy (exploratory cohort 1) and from control groups (active non-renal SLE patients (n = 25), patients with non-lupus glomerular diseases (n = 25) and healthy controls (25)) (initial screening stage). Results were also analyzed according to clinical outcome after treatment (responders (n = 22) vs. non-responders (n = 23)). Second, results were further confirmed in the validation cohort (cohort 2) that included 25 patients with active LN, of whom 16 achieved complete response during follow-up and 9 were non-responders (confirmation stage). Finally, urinary NRP-1 and VEGF levels were further measured three-monthly for at least 12 months in a new prospective cohort of patients with active LN (cohort 3) (n = 39). The study was approved by the Vall d'Hebron Ethic Committee and written informed consent was obtained from all patients.

1.3. Quantitative Reverse Transcription-PCR

For the quantification of mRNAs, 200 ng total RNA was reverse transcribed using the High Capacity RNA-to-cDNA Kit (Applied Biosystems) with the thermal cycler program: 25 °C for 5 min, 42 °C for 45 min and 82 °C for 5 min.

The mRNAs were quantified by TaqMan gene expression assays (FAM dye-labeled MGB probe, Applied Biosystems) using the ABI PRISM 7000 termocycler at 50 °C for 2 min, 95 °C for 10 min, followed by 40 cycles of 95 °C for 15 s and 60 °C for 1 min. The PCR primers for human NRP1 (Hs00826128_m1), for human VEGFA (Hs00900055_m1), human VEGFR1 (FLT1, Hs01052961_m1), human VEGFR2 (KDR, Hs00911700_m1) or human SEMA3A (Hs00173810_m1) were used (ThermoFisher). All cellular RNA samples were normalized based on the TaqMan Gene Expression Assays for human GAPDH (Hs02758991_g1). Relative changes in gene expression (fold change) were calculated using the $2^{-\Delta\Delta Ct}$ method.

1.4. Immunohistochemistry

Formalin-fixed paraffin-embedded tissue sections were deparaffinized underwent epitope unmasking by pressure-cooking in an EDTA solution (2 mM, pH = 8). Then the slides were incubated with H_2O_2 (3%) in methanol to block endogenous hydrogen peroxidase during 30 min at room temperature. Then, the samples were incubated with blocking buffer (0.1 M Tris-HCl/0.1%, BSA/0.1% Tween 20 (*v*/*v*)) during 1 h at room temperature and following they were incubated with corresponding primary antibody overnight at 4 °C: anti-human NRP-1 (sc-5307, Santa Cruz Biotechnology, 1:100), anti-VEGFA (sc-152, Santa Cruz Biotechnology, 1:250), anti-VEGFRI or anti-FLK1 (ssc-6251, Santa Cruz Biotechnology, 1:250) and anti-human SEMA3A (sc-28867, Santa Cruz Biothecnology, 1:100). Negative controls were obtained by substituting the primary antibody with a control irrelevant immunoglobulin G (IgG). Incubation with secondary antibodies conjugated with HRP (1:1000) were done during 1 hour at room temperature and after washing with PBS, the DAB substrate working solution was added and incubated by 15 min to obtain the staining.

As controls, we used disease-free kidney sections from tissue margins of total or subtotal nephrectomies obtained from patients undergoing surgery for renal malignancies.

1.4.1. Isolation of Primary T Cell Isolation from Healthy Donors

First, we isolated Peripheral Blood Mononuclear Cells (PBMCs) from total human blood by Ficoll gradient. To obtain a final volume of 5×10^7 PBMC, we obtained 4 tubes of 8 mL of total blood. After centrifugation (3000 rpm during 25 min at RT) we recovered the PBMCs. Second, we isolated Tcells by Dynabeads® Untouched™ Human T cells protocol *(*Invitrogen*)* following the manufacturer's protocol. This protocol is intended for isolation of untouched human T cells from PBMC by depleting B cells, NK cells, monocytes, platelets, dendritic cells, granulocytes, and erythrocytes. Finally, T cells were characterized using APC Mouse Anti-Human CD4 *(*BD Pharmingen™*)*, Anti-Human CD3 PE (Diaclone) and APC Mouse Anti-Human CD8 *(*BD Pharmingen™*)* by flow cytometry analyzer BD LSRFortessa™. The final step was the separation of CD3/CD4 and CD3/CD8 T cells by BD FACSAria II flow cytometer Cell Sorter *(*Biosciences).

1.4.2. Wound Healing Assay

To assess the potential angiogenesis of endothelial renal cells in different conditions (control and inhbited NRP-1) and stimulations, wound healing assays were performed. We used scratch assay in all the conditions. Firstly, 1×10^5 cells were plated in 24-well plate to obtain high confluence (70–80%). After that, the cell monolayer was scraped in a straight line to create a "scratch" with a p200 pipet tip. The derbis was removed and the edge of the scratch were smoothed by washing the cells with 1 mL of medium. To obtain the same field during the image acquisition, it is important to create markings to be used as references points close to the scratch. The cells were placed at 37 °C into the incubator and images were acquired at 0, 2, 4, 8, 12, and 24 h. To calculate migration, bright field microscopic images were analyzed using ImageJ software. The percentage of confluence index (CI) between the scratch was determined by the following equation:

$$\mathbf{CI} = \left(1 - \frac{\textit{wound distance at any time}}{\textit{wound distance at initial time}}\right) \times 100$$

When the distance between wound edges starts to be minor, the %CI between them will be higher. If there is not migration or wound healing in the scratch, the %CI will be around to zero value.

At least, three independent scratch experiments were performed in each condition.

1.4.3. Immunofluorescence Assay

For Immunofluorescence, cells were plated on glasses into 24-well plates. They were fixed using PFA 4% solution during 20 min at room temperature. After washing them with PBS, they were treated with 0.1% triton during 10 min. To block them, a solution of PBS 5% BSA was used during 1 hour at room temperature. After that, primary antibody rabbit anti-VEGFR2 (1:200, Santa Cruz Biotechnology) and mouse anti-NRP1 (1:200, Santa Cruz Biotechnology) were incubated overnight at 4 °C. As secondary antibodies, Alexa 488 donkey anti-rabbit (Abcam, ab150061) and Alexa 647 goat anti-mouse (Abcam, ab150119) were incubated for 2.5 h at room temperature (dilution 1:100). Fluoromount-G with DAPI (Invitrogen, Thermo Fisher Scientific, Carlsbad, CA) and Olympus BX61 motorized upright microscope with fluorescence were used to visualized the staining cells.

1.4.4. Evaluation of Immunohistochemistry

Results were evaluated on blinded specimens by the Vall d'Hebrón pathologist unit under the supervision of the nephropathologist (Dr. Marta Vidal). The percentage of cells expressing the different probes was scored semiquantitatively as follows: 0 (no expression), 1 (11–20%), 2 (40–60%), or 3 (>80%). Staining intensity was scored semiquantitatively as 0 (no staining), 1 (weakly positive), 2 (moderately positive), or 3 (strongly positive). This scores were obtained after the evaluation of 5 pathologists. After that, the mean of them has been expressed in the figures as "Average score".

2. Supplementary Figures

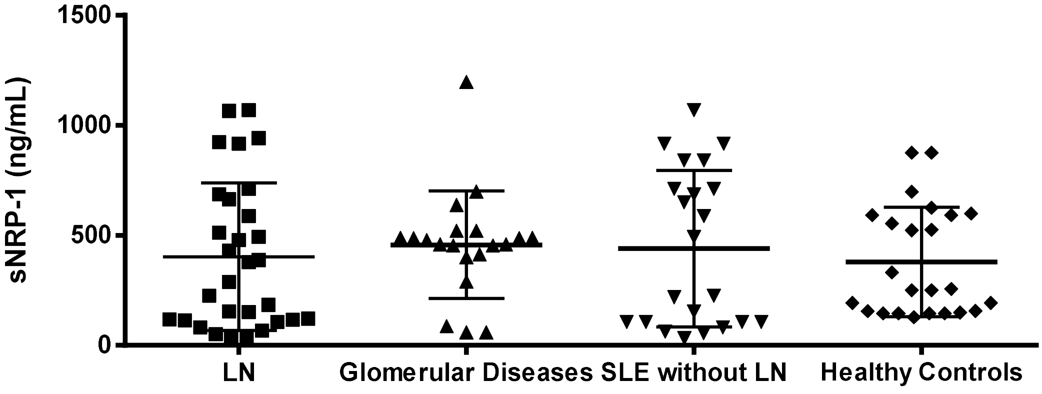

Figure S1. Protein level of sNRP-1 in the different cohorts.

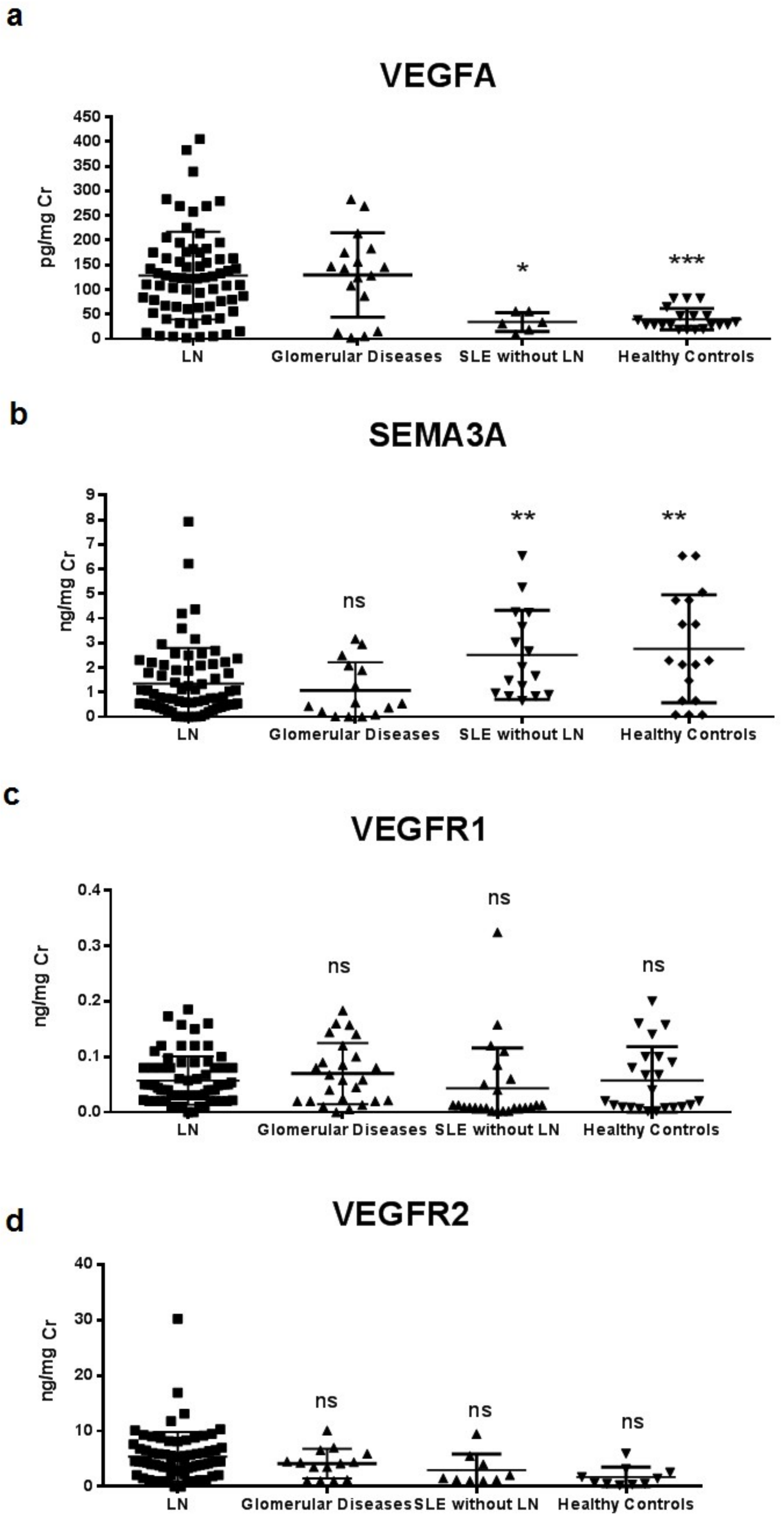

Figure S2. Protein level in the different study cohorts of: (**a**) uVEGFA (**b**) uSEM3A (**c**) uVEGFR1 and (**d**) uVEGFR2.

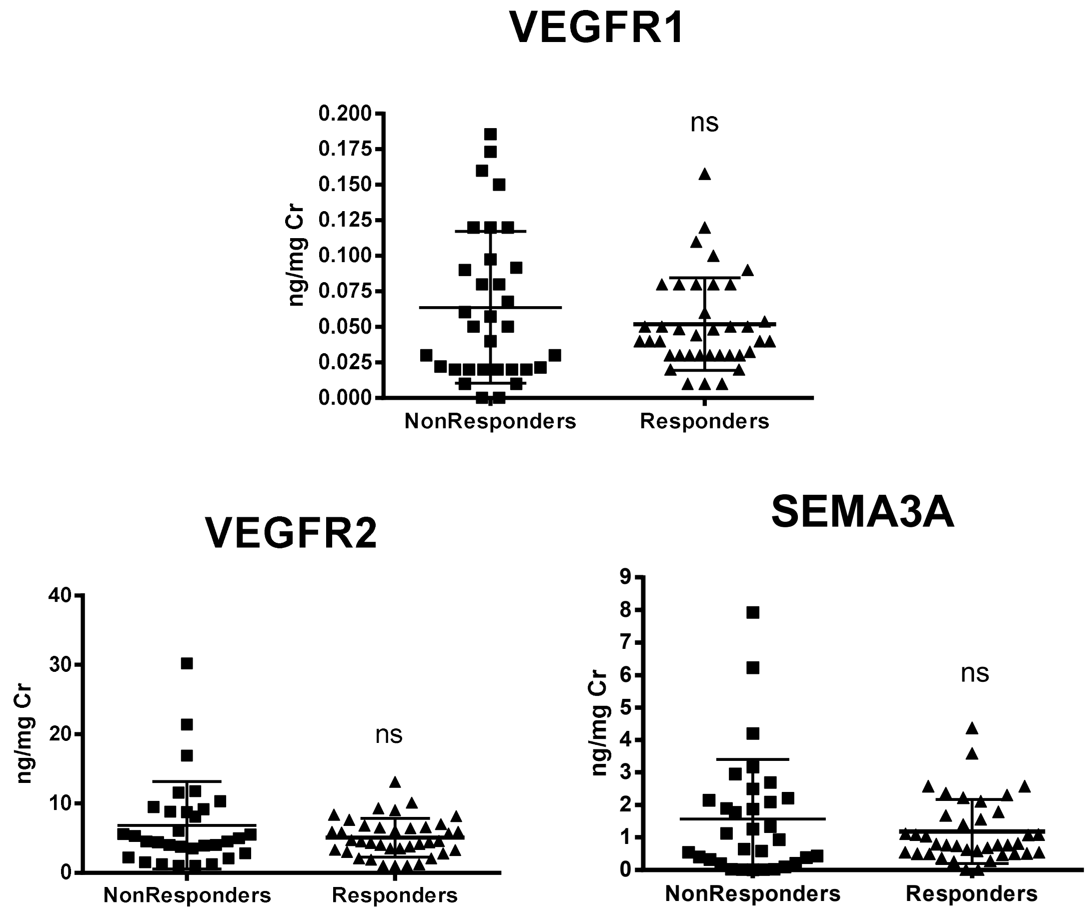

Figure S3. Protein level of uVEGFR1, uVEGFR2 and uSEM3A between Responders and NonResponders. Not significant differences were observed (ns).

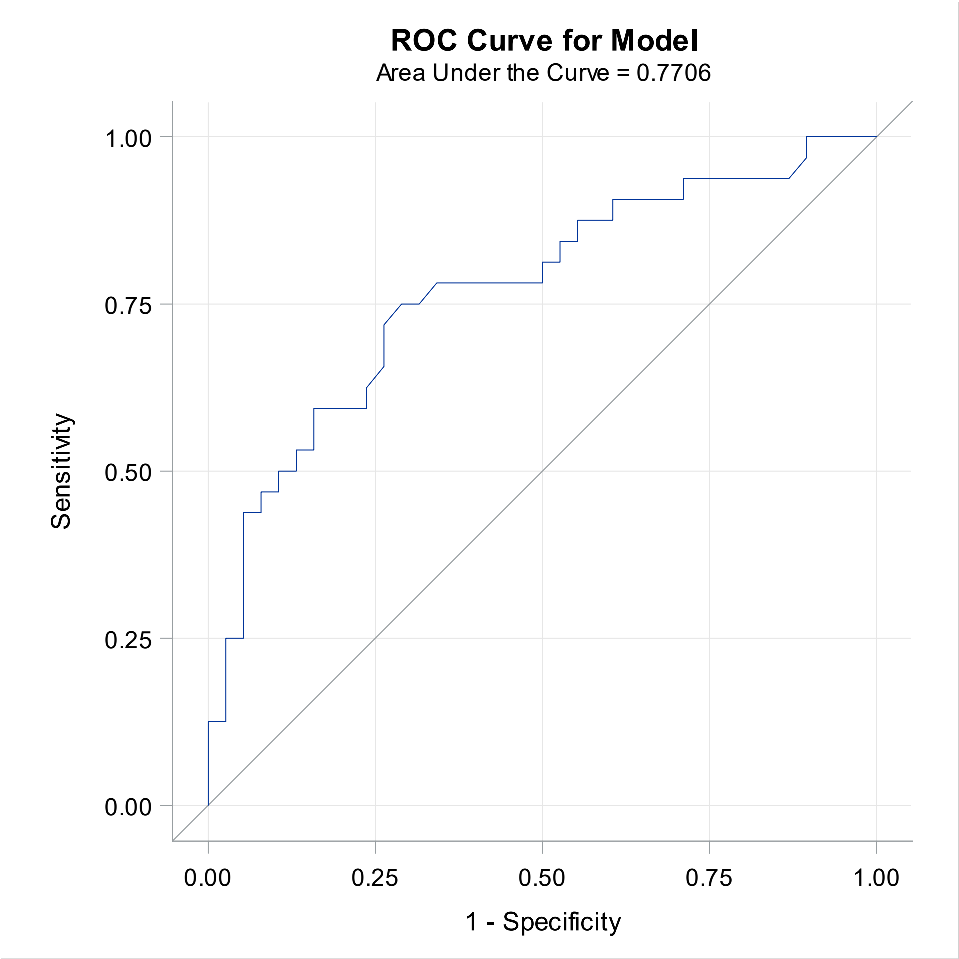

Figure S4. ROC curve Responders vs NonResponders (uVEGFA protein levels).

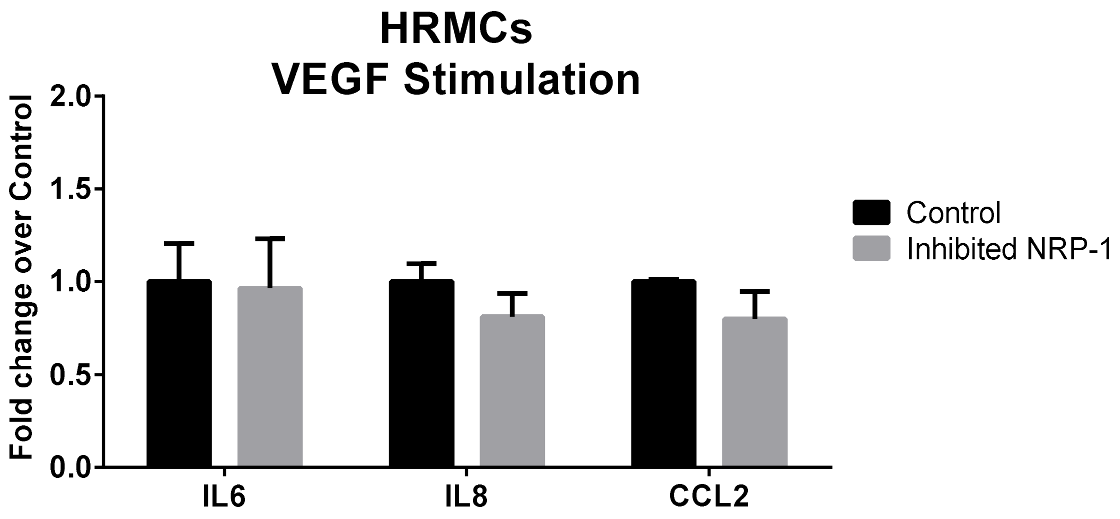

(**a**)

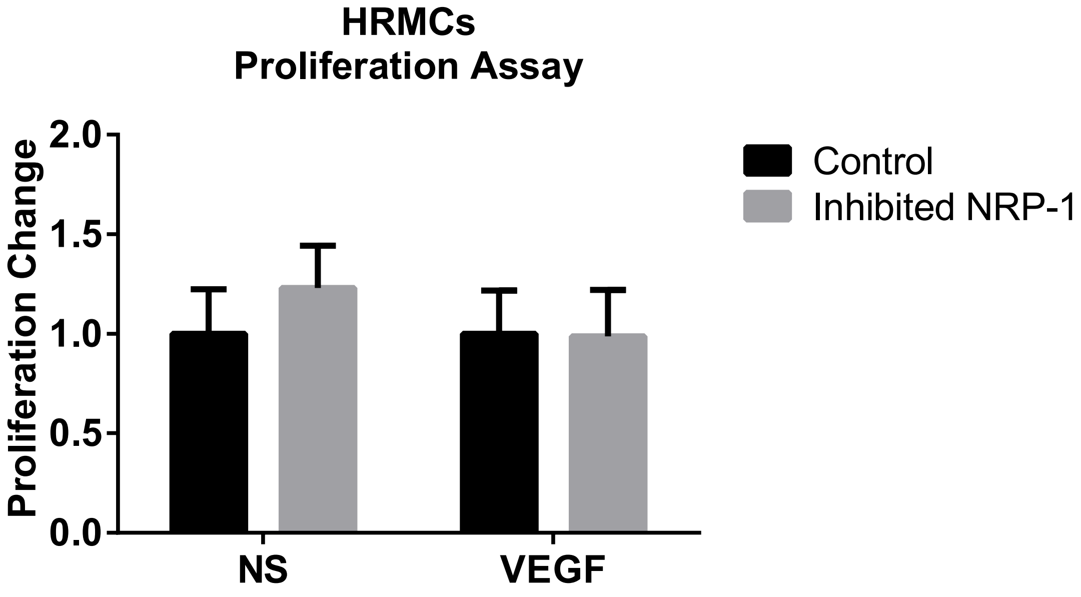

(**b**)

Figure S5. (**a**) Expression levels of IL6, IL8 and CCL2 were not difference between control and inhibited NRP-1 human renal mesangial cells (HRMCs). (**b**) Proliferation assay were similar between control and inhibited NRP-1 human renal mesangial cells (HRMCs) in both conditions (Non-Stimulated, NS, and VEGF stimulation).

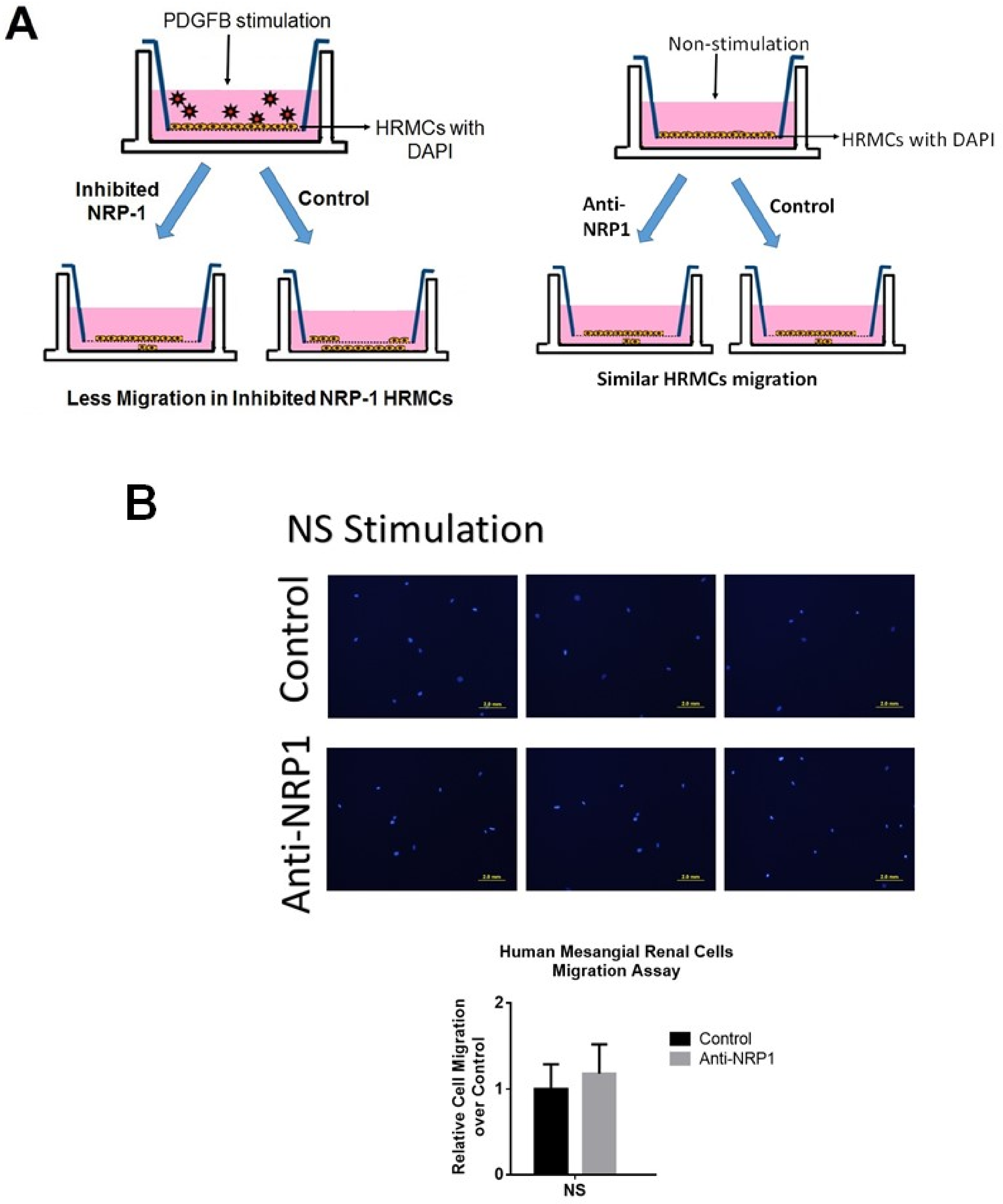

Figure S6. (**a**) Scheme of Transwell migration assay of HRMCs with PDGFB stimulation or in non-stimulation conditions. (**b**) Migration were similar between inhibited NRP-1 (anti-NRP1) and control cells in non-stimulation conditions.

3. Supplementary Table

Table S1. Clinical and histological variables at the time of the renal biopsy of the two cohorts and their combination.

Characteristics	Lupus nephritis (n = 70)			Active non-renal SLE (n = 25)	Other glomerular-diseases (n = 25)	Healthy Controls (n = 25)	p Value[a]
	Cohort 1 (n = 45)	Cohort 2 (n = 25)	Combined Cohort (n = 70)				
Demographic							

**Age, yrs**	40 ± 10	42 ± 11	41 ± 10	34 ± 6	35 ± 5	30 ± 4	0.4857<br>(0.787)<br>[0.885]
**Gender (Female/male)**	32/13	32/13	56/14	21/4	16/9	16/9	0.112<br>(0.723)<br>[0.112]
Race/ethnicity, n							
**White**	43	22	65	22	23	22	0.966<br>(0.966)<br>[0.500]
**Other**	2	3	5	3	2	3	
Laboratory parameters							
**Serum creatinine, mg/Dl**	1.1 ± 0.5	1.1 ± 0.5	1.1 ± 0.5	0.8 ± 0.1	1.9 ± 0.8	0.8 ± 0.3	0.035<br>(0.006)<br>[0.001]
**eGFR (mL/min)**	85 ± 29	77 ± 31	82 ± 30	104 ± 16	48 ± 36	95 ± 22	0.091<br>(0.001)<br>[0.006]
**Urea (mg/dL)**	52 ± 28	57 ± 29	54 ± 29	34 ± 14	81 ± 48	28 ± 11	0.076<br>(0.152)<br>[0.012]
**Anti-dsDNA Abs, IU/mL**	257 ± 62	318 ± 62	287 ± 68	39 ± 29	n.a.	n.a.	(0.087)
**Serum C3, mg/dL**	76 ± 33	79 ± 27	78 ± 31	94 ± 20	n.a.	n.a.	(0.101)
**Serum C4, mg/dL**	12.4 ± 9.4	13.5 ± 8.9	12.8 ± 9.2	13.4 ± 10	n.a.	n.a.	(0.090)
**Proteinuria, g/24 h**	3.3 ± 2.7	3.5 ± 3.4	3.3 ± 3.0	0.2± 0.1	9,1 ± 4,8	n.a.	(0.010)<br>[0.001]
**Leukocytes (cel/μL)**	128 ± 75	70 ± 105	95 ± 114	n.a.	n.a.	n.a.	
**Erythrocytes (cel/μL)**	123 ± 111	152 ± 141	133 ± 131	n.a.	n.a.	n.a.	
Disease index (SLEDAI-2K)							
**Total score**	14 ± 2	16 ± 1	16 ± 2	8 ± 2	n.a.	n.a.	(0.006)
**Renal score**	10 ± 2	13 ± 1	12 ± 1	0	n.a.	n.a.	
**Extra-Renal score**	4 ± 2	3 ± 2	4 ± 2	8 ± 2	n.a.	n.a.	(0.001)
Renal Flare, n							
**Proteinuric**	37	22	59	n.a.	n.a.	n.a.	
**Neprhitic**	8	3	11	n.a.	n.a.	n.a.	
**Debut**	26	15	41	n.a.	n.a.	n.a.	
**Relapsing**	19	10	29	n.a.	n.a.	n.a.	
Renal Biopsy, n (%)							
**Class III**	4 (9)	5 (20)	9 (13)	n.a.	n.a.	n.a.	
**Class IV**	36 (80)	17 (68)	53 (76)	n.a.	n.a.	n.a.	
**Class V**	5 (11)	3 (12)	8 (11)	n.a.	n.a.	n.a.	
**Activity Index**	7.5 ± 4.0	7.5 ± 2.4	7.5 ± 3.6	n.a.	n.a.	n.a.	
**Chronicity Index**	1.7 ± 1.7	2.4 ± 2.2	2.0 ± 1.9	n.a.	n.a.	n.a.	

Values are means ± SE. BUN, blood urea nitrogen; eGFR, estimated glomerular filtration rate; anti-dsDNA, anti-double-stranded DNA; n.a., not applicable. [a]*P*-value refers to the comparison of the LN with healthy controls, the values in bracket referred to the comparison with active non-renal SLE and the values in square bracket referred to the comparison with other glomerular-disease cohort: Mann-Whitney U Test or Pearson $\chi 2$ test.

Table S2. Baseline characteristics of patients from the two cohorts and combining the two cohorts according to immunosuppressive therapy.

**Characteristics**	**Responders**			**No-Responders**			***p* Value**
	**Cohort 1 (*n* = 22)**	**Cohort 2 (*n* = 16)**	**Combined Cohort (*n* = 38)**	**Cohort 1 (*n* = 23)**	**Cohort 2 (*n* = 9)**	**Combined Cohort (*n* = 32)**	
Demographic							
**Age (years)**	38 ± 9	42 ± 10	42 ± 12	42 ± 12	41 ± 9	39 ± 8	0.517

**Gender (Female/male)**	13/9	14/2	33/5	19/4	8/1	27/5	
Race/ethnicity, n							
**White**	21	15	35	22	8	30	0.811
**Hispanic**	1	1	3	1	2	2	
Laboratory parameters							
**Serum creatinine, mg/dL**	1.0 ± 0.3	1.0 ± 0.5	1.0 ± 0.4	1.3 ± 0.6	1.3 ± 0.6	1.3 ± 0.6	0.025
**eGFR (mL/min)**	89 ± 22	87 ± 31	88 ± 26	81 ± 36	71 ± 25	75 ± 33	0.167
**Urea (mg/dL)**	46 ± 24	50 ± 31	48 ± 27	58 ± 32	67 ± 25	61 ± 30	0.054
**Anti-dsDNA Abs, IU/mL**	332 ± 82	315 ± 91	344 ± 84	176 ± 60	259 ± 71	220 ± 68	0.781
**Serum C3, mg/dL**	76 ± 36	77 ± 28	77 ± 32	77 ± 30	82 ± 27	79 ± 29	0.878
**Serum C4, mg/dL**	11.4 ± 10.1	13.0 ± 8.9	12.0 ± 9.6	13.5 ± 8.8	14,3 ± 9.5	13.7 ± 8.8	0.248
**Proteinuria, g/24 h**	3.5 ± 3.4	4.0 ± 3.85	3.0 ± 3.2	3.1 ± 2.0	3.2 ± 3.2	3.4 ± 2.7	0.563
**Leukocytes (cel/µL)**	90 ± 120	97 ± 110	95 ± 114	111 ± 155	99 ± 185	101 ± 190	0.495
**Erythrocytes (cel/µL)**	103 ± 101	157 ± 121	133 ± 131	157 ± 297	201 ± 297	187 ± 397	0.158
Disease index (SLEDAI-2K)							
**Total score**	15 ± 2	13 ± 3	14 ± 3	14 ± 1	15 ± 2	15 ± 2	0.145
**Renal score**	11 ± 1	9± 2	10 ± 2	10 ± 2	9 ± 3	9 ± 3	0.112
**Extra-Renal score**	4 ± 2	5 ± 2	5 ± 3	4 ± 2	4 ± 2	4 ± 2	0.184
Renal Flare							
**Proteinuric**	22	16	38	8	6	14	<0.0001
**Nephritic**	0	0	0	15	3	18	<0.0001
**Debut**	16	11	27	10	4	14	0.021
**Relapsing**	8	5	11	13	5	18	0.021
Renal Biopsy, n (%)							
**Class III**	2 (9)	2 (12)	4 (11)	2 (9)	1 (11)	3 (9)	0.714
**Class IV**	18 (82)	12 (76)	29 (76)	19 (82)	7 (78)	26 (82)	0.618
**Class V**	2 (9)	2 (12)	5 (13)	2 (9)	1 (11)	3 (9)	0.441
**Activity Index**	6.7± 3.4	8.7± 1.0	7.4± 3.2	8.0 ± 4.4	6.0 ± 2.5	7.6 ± 4.0	0.864
**Chronicity Index**	1.4 ± 2.3	2.2 ± 1.9	1.7 ± 2.1	1.8 ± 1.3	3.6 ± 2.8	2.2 ± 1.8	0.203

Values are expressed by means ± standard deviation (SD). eGFR, estimated glomerular filtration rate; anti-dsDNA, anti-double-stranded DNA (reference range <15 UI/mL). *p*-value refers to the comparison of the Responders with Non-Responders in combined cohorts by Mann-Whitney U Test or Pearson $\chi2$ test.
